# Medical Faculty and Medical Student Opinions on the Utility of Questions to Teach and Evaluate in the Clinical Environment

**DOI:** 10.1007/s40670-023-01780-5

**Published:** 2023-04-11

**Authors:** Lloyd Rucker, Garrett Rucker, Angelica Nguyen, Maria Noel, Maria Marroquin, Elani Streja, Eileen Hennrikus

**Affiliations:** 1 Department of Medicine, Irvine School of Medicine, University of California, 101 the City Drive Orange, Irvine, CA 92868 USA; 2 Tibor Rubin Long Beach Veterans Administration Hospital, Long Beach, CA USA; 3 Department of Medicine, MedStar Washington Hospital Center – Georgetown University, Washington, DC USA; 4 Irvine School of Medicine, University of California, Irvine, CA USA; 5 Department of Emergency Medicine, Christiana Care, Newark, DE USA; 6 California State University, Northridge, Northridge, CA USA; 7 Division of Nephrology, Department of Medicine, University of California, Irvine School of Medicine, Irvine, CA USA; 8 Penn State University School of Medicine, Hershey, PA USA

**Keywords:** Undergraduate medical education, Questions, Pedagogy, Wellness, Pimping

## Abstract

**Objectives:**

We sought to report medical student and faculty perceptions of the purpose and utility of questions on clinical rounds.

**Methods:**

We developed and administered a survey to third and fourth-year medical students and teaching physicians. The survey elicited attitudes about using questions to teach on rounds in both benign and malignant learning environments.

**Results:**

Ninety-seven percent of faculty and 85% of students predicted they will use questions to teach. Nine percent of students described learning-impairing stress during benign bedside teaching. Fifty-nine percent of faculty felt questions were mostly for teaching; 74% of students felt questions were mostly for evaluation. Forty-six percent of students felt questions underestimated their knowledge. Students felt questions were more effective for classroom teaching than bedside teaching. Faculty and students agreed that a malignant environment detrimentally affected learning and performance.

**Conclusions:**

Students and faculty supported the use of questions to teach and evaluate, especially in benign teaching environments. Many students described stress severe enough to affect their learning and performance, even when questioned in benign teaching environments. Faculty underestimated the degree to which students experience stress-related learning impairment and the degree to which students see questions as evaluation rather than teaching. Nearly half of students felt that questions underestimated their own knowledge. Students feel more stress and less learning when questioned with a patient present. Faculty must realize that even in the best learning environment some students experience stress-impaired learning and performance, perhaps because of the conflict between learning and evaluation.

## Introduction

In 1916, Abraham Flexner recorded this admiring observation of William Osler on bedside rounds: “Rounded with Osler today. Riddles house officers with questions. Like a Gatling Gun. Welch says students call it pimping. Delightful” [1]. Medical faculty almost universally use questions to teach and evaluate students, with much the same enthusiasm for the practice as Flexner expressed. In one survey, 97% of internal medicine clerkship faculty agreed that questions were “valuable” for education [2].

In the past, rather than focus on defining the actual benefits and risks of questions to explore understanding and enhance critical thinking, medical education researchers and commentators have focused on the potentially hostile environment created by using questions to teach, a practice some refer to as “pimping” [1, 3–7]. This focus on “pimping” has diverted attention from the relative benefits and risks of various questioning styles and venues.

The learning environment of rounds is chaotic and high stakes, even when attendings strive to make it less so. Questions can potentially both facilitate learning and cause humiliation. Indeed, students have described humiliation both as “abusive” and as effective pedagogy [8–11]. But just as humiliation *may* be a stimulant to learning for some students, it is also possible that well-structured and formative questions can cause significant stress for other students, impairing their ability to learn and impeding their ability to “show their stuff” on rounds. Some, but not all, questions stimulate thought, explore understanding, challenge paradigms, and identify gaps in knowledge. Not all students and not all questions are the same.

Sometimes, faculty and students justify pimping or humiliation as Socratic, [12, 13], but not all questions are either truly Socratic or effective pedagogy. Socrates used questions to explore big issues, calling on his students to think broadly, to overcome bias and prior assumptions, to breakdown beliefs and put them back together again, and to challenge what was “known.” Plato reported that Socrates did not generally ask narrow and fact-based questions because he believed that facts and beliefs should be challenged.

We know from prior work that many students and faculty feel the use of questions in clinical teaching situations stimulates learning [2, 7–11]. We also know that most students have experienced or observed humiliating questioning [7, 8, 11]. However, prior research has evaluated student attitudes in general; it is not clear how individual students feel about the effect of questioning on their own level of stress or their personal ability to learn and perform on rounds. An individual student might feel that questions are generally useful for learning, but that for himself or herself personally, questions induce learning-impairing stress. Understanding the risks as well as the benefits of questions as a teaching tool on clinical services begins with an understanding of teachers’ and students’ attitudes and beliefs about the utility of questions.

We developed a survey tool to explore medical student and faculty beliefs about the use of questions to teach and evaluate. We sought to clarify opinions on the use of questions to teach and evaluate and to contrast student and faculty opinions. The specific issues we sought to clarify were the following:

1. To what degree do students and faculty agree upon and support the use of questions to teach and evaluate on the clinical teaching services?
2. What do students and faculty feel are the goals of using questions for teaching or for evaluation?
3. To what degree do students and faculty feel the use of questions causes stress that impairs learning or performance?
4. Do students and faculty feel questions on rounds can accurately assess students’ knowledge or skills?
5. How do students and faculty feel the learning environment (benign versus malignant) and the learning venue (classroom versus bedside) affect the stress caused by questions?

## Materials and Methods

### Surveys

We developed surveys to define student and faculty attitudes. We employed an iterative evaluation process to construct our questions. We constructed questions in a parallel fashion with faculty and student questions each addressing the same attitude or belief. The study consisted of 22 Likert or single response questions. We asked faculty to answer the first 8 questions from their point of view as teachers. However, to examine faculty beliefs about how students experience questions on rounds, we asked the faculty to answer the subsequent 14 items from the point of view of a junior medical student. That is, we asked the faculty what they *imagined* the typical third-year medical student would feel or believe. This allowed us to compare what students said they felt to what faculty thought students would say they felt. The survey compared attitudes about questions as teaching tools and as evaluation tools, commitment to the use of questions, and the degree and effect of stress created by questions in various environments and venues. We presented scenarios that represented the best possible case and the worst possible case, both in the classroom and at the bedside. We recognized that these represented the extremes and that most situations would fall between the extremes.

Some consider “pimping” to include the use of any questions in any real-time clinical learning environment, no matter the intent or tone of the questions. Others use the term pimping only when teachers use questions to harass or humiliate. Because of the ambiguous and fraught nature of the term, we avoided it entirely in our surveys and chose to characterize the learning environment in explicit terms.

We defined a benign questioning environment when faculty asked specific, reasonable, well-structured, and relevant questions that should be answerable and that were asked in a calm, supportive, and unintimidating style.

We defined a malignant questioning environment when faculty asked difficult and marginally relevant questions in an intimidating fashion with rapid fire questions until the learner could no longer find answers.

A PhD epidemiologist (ES) with training and experience in questionnaires helped write and review the survey questions. To refine and validate the survey questions, we then presented the proposed questions to 21 third-year medical students and 10 experienced clinical teachers. To assess validity, we asked the following questions:

1. Do the situations describe the learning environment that includes the extremes of possible types of questioning and specific situations that occur on clinical teaching rounds?
2. Do the outcomes described include the extremes of the effects of the use of questioning on rounds on learner’s ability to learn on rounds and their incentive to learn after rounds?
3. Do the extremes of questioning described reflect your own experience or the experience of others that you have directly observed?
4. Do you feel the survey questions effectively measure the effects of using questions to teach and evaluate?
5. Do you feel the survey questions effectively measure the degree of stress experienced by students?
6. Do you fully understand the situations described and the terms used?

We evaluated questions through several rounds of feedback until all evaluators felt that the questions were clear and addressed our stated goals. None of the responses from the validation study were used in the final data set, and none of the students or faculty in the validation set participated in the study. The time to complete the study was about 10 min.

### Setting

We conducted the study at the University of California, Irvine School of Medicine in Irvine, California (UCI), and at the Penn State School of Medicine in Hershey, Pennsylvania (PSU). We distributed the survey between May 1, 2020, and August 4, 2020, in both paper and online formats.

### Participants

We surveyed medical students in their fourth year at UCI or their third and fourth years at PSU. All responses were anonymous. We surveyed faculty teachers from hospitalist internal medicine, internal medicine primary care, and general surgery. We did not exclude any faculty or students who met these basic criteria.

### Data Analysis

We reported survey responses according to faculty or student status as median (interquartile range) and percentage of each response type and compared them using Mann Whitney *U* or Fisher’s exact test. We similarly compared students’ responses about their ability to learn at the bedside and in the classroom and in both benign and malignant environments, as defined above. Statistics were run with Stata. Results were reported as agreed if there was an absence of statistical significance in difference of response distribution at *p* < 0.05. For instance, we compared student responses to faculty responses or student responses in one teaching environment to the same students’ responses in a different teaching environment or venue.

### Institutional Review Board Approval

Institutional review boards at both sites approved the study as exempt.

## Results

Table 1 shows the demographic information collected on participants. One hundred eleven faculty (45.5% of invited faculty participants) and 224 students (57.3% of invited student participants) completed the surveys. Student participants were members of the classes of 2021 at UCI and 2021 and 2022 at PSU.

**Table 1:** Respondents

	Total #	Male %	Years teaching # (%)			Discipline # (%)		
Faculty	111	54	1–5	6–15	> 15	Primary care	Hospitalist	General surgery
			47 (42)	35 (32)	29 (26)	10 (9)	81 (73)	20 (18)
Students	224	43						

Table 2 compares faculty and student opinions on the utility of questions to teach and to evaluate. Students were more likely than faculty to believe that faculty use questions for evaluation rather than or in addition to teaching (*p* < 0.001). Fifty-nine percent of faculty felt that questions were used mostly or only to teach; 26 percent of students felt that questions were used mostly or only to teach. Seventy-four percent of students felt that questions were used for equal measure teaching and evaluation or mostly to evaluate; that is, 74% of students felt faculty evaluation of student performance was as important or more important a goal than teaching.

**Table 2:** Opinions on the effectiveness of questions to teach and evaluate when faculty and students answer from their own perspective, percent

1. Attending teachers ask questions during clinical teaching rounds						Median (IQR)	MW
	Entirely to Evaluate	Mostly to Evaluate	Equal Measure	Mostly to Teach	Entirely to Teach		
Faculty	0	1	40	54	5	4(3,4)	< 0.001
Students	2	12	60	25	1	3(3,4)	
2. When used as an evaluation tool, questions are reliable and effective to evaluate the learner’s factual medical knowledge base							
	Strongly Disagree	Disagree	Neutral	Somewhat Agree	Strongly Agree		
Faculty	1	8	22	53	16	4(3,4)	0.34
Students	2	12	20	53	13	4(3,4)	
3. When used as an evaluation tool, questions are reliable and effective to evaluate the learner’s clinical reasoning skills							
	Strongly Disagree	Disagree	Neutral	Somewhat Agree	Strongly Agree		
Faculty	0	2	14	52	32	4(4,5)	0.04
Students	1	5	15	57	21	4(4,4)	
4. When used as an evaluation tool, questions are reliable and effective to evaluate the learner’s management knowledge							
	Strongly Disagree	Disagree	Neutral	Somewhat Agree	Strongly Agree		
Faculty	0	3	12	62	23	4(4,4)	< 0.001
Students	0	6	26	58	9	4(3,4)	
5. A teacher’s asking questions during clinical rounds is effective to facilitate or enhance learning *during* rounds							
	Strongly Disagree	Disagree	Neutral	Somewhat Agree	Strongly Agree		
Faculty	0	1	8	45	46	4(4,5)	< 0.001
Students	2	5	19	43	30	4(3,5)	
6. A teacher’s asking questions during clinical rounds is effective to stimulate learning and reading *after* rounds							
	Strongly Disagree	Disagree	Neutral	Somewhat Agree	Strongly Agree		
Faculty	0	6	14	39	31	4(4,5)	0.97
Students	1	5	11	44	38	4(4,5)	
7. How likely are you to use questions on clinical rounds in the future to teach?							
	Will not use questions	Not likely to use questions	Neutral Not certain	Somewhat likely to use questions	Very likely to use questions		
Faculty	0	1	3	22	75	5(4,5)	< 0.001
Students	0	3	11	40	45	4(4,5)	
8. How likely are you to use questions on clinical rounds in the future to evaluate learner’s knowledge?							
	Will not use questions	Not likely to use questions	Neutral Not certain	Somewhat likely to use questions	Very likely to use questions		
Faculty	0	8	11	34	47	4(4,5)	< 0.001
Students	2	10	30	35	22	4(3,4)	

Faculty (69%) and students (66%) on balance agreed that questions reliably evaluate factual medical knowledge (*p* = 0.34).

Faculty (85%) and students (67%) agreed that questions were useful to evaluate management skills, but faculty felt more strongly that questions were useful for these evaluations (*p* = 0.04 and *p* ≤ 0.001, respectively).

While generally agreeing that questions were useful to stimulate learning during rounds, faculty (91%) felt more strongly than students (73%) that questions were useful to do so (*p* ≤ 0.001).

Both faculty (97% to teach; 81% to evaluate) and students (85% to teach; 57% to evaluate) predicted that they would use questions to teach and to evaluate in the future. However, faculty felt much more strongly than students that they would do so (*p* ≤ 0.001 and *p* ≤ 0.001, respectively, for teaching and evaluation).

Table 3 compares the opinions students declared to the opinions that faculty believed students would declare. The survey responses addressed opinions about the way questions affect learning and performance in benign question environments and in malignant question environments, either in the classroom during attending rounds or at the bedside during attending rounds with a patient present. Majorities of both students and faculty expressed the opinion that students felt a question asked in a benign question environment has positive effects on learning and the ability to demonstrate knowledge and skills. For instance, 98% of students and 90% of faculty felt that a question posed in the classroom in a benign learning environment would have a positive effect on learning.

**Table 3:** Students’ actual opinions and teachers’ perception of students’ opinions on the effect of benign and malignant questioning styles on ability to learn, on ability to demonstrate knowledge and on students’ stress levels in the classroom or at the bedside in front of patients, percent

	− 4 Dramatic negative effect	− 3	− 2	− 1	0	+ 1	+ 2	+ 3	+ 4 Dramatic positive effect	Median (IQR)	MW
1. In a classroom, benign question effect on my ability to learn											
Faculty*	0	0	0	2	8	11	24	27	28	3(2,4)	< 0.001
Students	0	0	1	0	1	8	24	24	42	3(2,4)	
2. In a classroom, benign question effect on my ability to demonstrate knowledge or skills											
Faculty*	0	0	3	0	2	11	28	27	29	3(2,4)	0.12
Students	0	0	1	0	0	9	23	32	34	3(2,4)	
3. At the bedside with a patient present, benign question effect on my ability to learn											
Faculty*	0	1	2	8	5	11	30	21	22	2(1,3)	0.24
Student	1	0	3	5	3	9	24	26	26	3(2,4)	
4. At the bedside with a patient present, benign question effect on my ability to demonstrate knowledge or skills											
Faculty*	1	1	6	5	3	15	24	23	23	2(1,3)	0.41
Student	1	0	5	6	4	11	20	29	24	3(1,3)	
5. In a classroom, malignant question effect on my ability to learn											
Faculty*	35	16	18	12	6	9	3	2	0	− 3(− 4, − 1)	0.08
Student	29	11	19	16	6	9	5	4	1	− 2(− 4, − 1)	
6. In a classroom, malignant question effect on my ability to demonstrate knowledge or skills											
Faculty*	32	19	19	12	4	8	5	1	0	− 3(− 4, − 1)	0.17
Student	30	12	20	15	7	7	5	3	1	− 2(− 4, − 1)	
7. At the bedside with a patient present, malignant question effect on my ability to learn											
Faculty*	46	17	13	12	4	5	2	2	0	− 3(− 4, − 2)	0.72
Student	46	14	17	7	4	5	5	3	0	− 3(− 4, − 2)	
8. At the bedside with a patient present, malignant question effect on my ability to demonstrate knowledge or skills											
Faculty*	49	12	14	10	5	6	2	2	0	− 3(− 4, − 1.5)	0.57
Student	43	17	18	8	3	4	4	2	1	− 3(− 4, − 2)	
9. Students Only: Ability to learn, benign question environment											
In Classroom	0	0	1	0	1	8	24	24	42	3(2,4)	< 0.001
At Bedside	1	0	3	5	3	9	24	26	26	3(2,4)	
10. Students Only: Ability to demonstrate knowledge or skills, benign question environment											
In Classroom	0	0	1	0	0	9	23	32	34	3(2,4)	< 0.001
At Bedside	1	0	5	6	4	11	20	29	24	3(1,3)	
11. Students Only: Ability to learn, malignant question environment											
In Classroom	29	11	19	16	6	9	5	4	1	− 3(− 4, − 1)	< 0.001
At Bedside	46	14	17	7	4	5	5	3	0	− 3(− 4, − 2)	
12. Students Only: Ability to demonstrate knowledge or skills, malignant environment											
In Classroom	30	12	20	15	7	7	5	3	1	− 3(− 4, − 2)	< 0.001
At Bedside	43	17	18	8	3	4	4	2	1	− 3(− 4, − 2)	

*Faculty response from what they believed to be the student’s perspective

Majorities of students and faculty supported the opinion that students believe a question asked in a malignant question environment has negative effects on learning and on a student’s ability to demonstrate knowledge and skills. For instance, 84% of students and 88% of faculty felt that questions asked in a malignant environment at the patient bedside would have a negative effect on learning. These opinions were not universal: thirteen percent of students and 9% of faculty felt that questions asked in a malignant question environment at the bedside would have a *positive* effect on learning. Students (98%) expressed the opinion that a benign question environment had positive effects on learning significantly more strongly than faculty (90%) anticipated they would (*p* ≤ 0.001). No additional comparisons between students and faculty were statistically different.

Also in Table 3, when comparing students’ opinions about questions asked in benign classroom or benign bedside settings, students expressed more positive opinions of the effect of questions asked in the classroom (98% positive for learning, 99% positive for evaluation) than questions asked at the bedside (88% positive for learning, 84% positive for evaluation) both on learning (*p* ≤ 0.001) and on demonstrating knowledge and skills (*p* ≤ 0.001). When comparing students’ opinions about questions asked in malignant classroom and bedside settings, students expressed more negative opinions on the effect of questions on learning when at the bedside (84% negative) than when in the classroom away from patients (75% negative) (*p* ≤ 0.001).

Table 4 shows faculty correctly estimated that students generally felt that questions on clinical rounds were not an accurate reflection of the student’s knowledge base (*p* = 0.10). Faculty underestimated the degree to which students felt questions underestimated their clinical reasoning skills (*p* = 0.03). Forty-six percent of students felt that questions on rounds underestimated their own, personal knowledge base; 36% of students felt that questions on rounds underestimated their own, personal clinical reasoning skills.

**Table 4:** Students’ opinions and teachers’ perception of students’ opinions on whether responses to questions are an accurate reflection of knowledge base and clinical reasoning skills, percent

	− 4 Dramatically underestimates	− 3	− 2	− 1	0	+ 1	+ 2	+ 3	+ 4 Dramatically overestimates	Median (IQR)	MW
1. To what degree do you feel that your responses to questions from attendings on clinical rounds were an accurate reflection of your knowledge base?											
Faculty*	5	6	5	16	41	13	14	2	0	0(− 1,1)	0.10
Students	3	4	16	23	30	10	11	3	1	0(− 1,0)	
2. To what degree do you feel that your responses to questions from attendings on clinical rounds were an accurate reflection of your clinical reasoning skills?											
Faculty*	4	4	5	11	41	12	16	7	0	0(0,1)	0.03
Students	3	4	11	18	38	10	11	5	0	0(− 1,1)	

*Faculty response from what they believed to be the student’s perspective

Table 5 shows faculty tended toward underestimating the degree of stress that students expressed in benign question environments (*p* = 0.07) and correctly estimated the significant degree of stress students felt in malignant question environments (*p* = 0.82). Even in a benign question environment at the bedside, 9% of students experienced stress that they felt impaired their learning ability.

**Table 5:** Perception of personal stress level when asked benign or malignant questions, percent

	1	2	3	4	5	6	7	8	9	Median (IQR)	MW
	No Stress		Mild stress		Moderate stress but functional		Severe Stress		Highest stress Cannot function		
1. Personal stress level with benign questions											
Faculty*	9	11	36	8	29	7	0	0	0	3(3,5)	0.07
Students	6	10	30	10	33	8	3	0	0	4(3,5)	
2. Personal stress with malignant questions											
Faculty*	1	1	8	5	19	16	30	9	11	6.5(5,7)	0.82
Students	0	1	3	8	18	19	30	13	7	7(5,7)	

*Faculty response from what they believed to be the student’s perspective

Table 6 shows faculty and students agreed that the best environment for teaching was an equal mix of teaching with and without questions (*p* = 0.12). However, 30% of students felt that they learned better on teaching rounds when faculty asked few or no questions, while 23% of faculty felt that students would say students learned better when few or no questions were asked during teaching rounds.

**Table 6:** Mix of teaching with and without questions for the best learning environment, percent

I learn better on clinical attending rounds when attendings use the following mix of questions and direct teaching without questions										Median (IQR)		Fisher’s exact *p* value
	No questions		Few questions		Equal mix		Mostly questions		All questions			
Faculty*	1		22		55		22		0	3(3,3)		0.12
Students	6		24		50		19		1	3(2,3)		
If the ward attending was only responsible for teaching and had no responsibility for evaluation, would that lead to a better learning environment and more effective teaching?												
		Yes		No change		Worse		No opinion				
Faculty		26		44		17		13			< 0.001	
Students		51		23		15		11				

*Faculty response to what they believed to be the student’s perspective

Twenty-six percent of faculty and 51% of students felt that if the ward attending did not have responsibility for evaluation, this would lead to a better learning environment (*p* ≤ 0.001 for the difference between students and faculty).

## Discussion

Medical teachers, from freshly minted medical students to seasoned faculty, use questions to teach. Ironically, in a discipline that teaches and honors its evidence base, little empiric evidence exists that questions are an effective pedagogical tool for teaching medicine in clinical settings and even less evidence exists about the best way to use questions [4]. This was a study of attitudes, perceptions, and beliefs. It was not a study of actual benefits or harms. We did not measure actual learning outcomes or stress levels. However, these findings can certainly provide guidance for faculty teachers as well as topics for further research.

In an opinion piece cited by the American College of Physicians as one of the most important articles of 2022, Kinnear et al. argued that questions for teaching are most effective when teachers create a supportive educational environment by (1) examining their motivations for teaching; (2) eliminating strategies aimed at reinforcing hierarchy, creating fear, or humiliating learners; and (3) teaching within a framework of educational safety [14]. Pylman and Ward have recently offered 12 tips for effective questioning in medical education and argued that more attention should be paid to reinforcing the right way to teach as opposed to criticizing the wrong way to teach [15]. The consensus seems to be that so long as questions are asked in the right way in a supportive educational environment, they have a positive effect on learning. This is certainly intuitively plausible despite the dearth of empiric evidence. To paraphrase Carl Sagan, the absence of evidence is not evidence of the absence of the effectiveness of questions. However, moving forward, as an evidence-based discipline, we should be researching actual outcomes of the learning and evaluation process for clinical medical education.

Questions as a teaching tool are here to stay. Our survey demonstrated the significant degree to which both students and faculty believed in and supported the use of questions to teach and to evaluate on clinical rotations. Ninety-seven percent of faculty and 85% of students said they will use questions to teach in the future. These findings supported the view that teaching and evaluating with questions is part of the culture of medical education and deeply ingrained in practice.

Reason exists to be hopeful about the utility of questions to teach in clinical medicine. Certainly, the strong support from students and teachers is one important endorsement. Faculty and students expressed the same opinion that a benign question environment enhances learning and is useful for evaluation. In fact, as in the work of Abbou-Hanna [2], faculty in our study underestimated the degree to which students supported the use of questions to teach in a benign question environment.

Research from other disciplines supports the use of questions to enhance learning. Broad-based education research in settings other than clinical medicine teaching rounds supports the use of testing with questions to enhance learning [16]. Well-constructed questions effectively catalyze successful learning and enhance retention [16]. Test-enhanced learning and reflection may also promote long-term retention in medical education [17]. However, at this time, we must be forthright that we lack significant evidence that questions posed in the unique environment of the clinical medical teaching service enhance learning. We also don’t know if questions on rounds validly measure knowledge or skills.

Our work suggests that reality may be more nuanced than the belief that everything always works well if well-trained and positive faculty use good questions in supportive environments. Our data suggest that there is good reason for faculty to be cautious and remain aware. The nuances we see are (1) the unrecognized stress induced by questions even when asked in a benign question environment; (2) the potential negative effects of the dual role of questions for both teaching and evaluation in the high stakes environment of clinical medical education; (3) the differences in effectiveness when teaching occurs at the bedside as opposed to the classroom; and (4) the persistent belief of some teachers and students that harsh or “toxic” quizzing stimulates learning.

Meaningful numbers of students in our survey experienced stress, which they felt negatively affected their learning and performance. Most comments in the literature on the stress induced by questions have been related to toxic questions in malignant environments. However, our work documents that even in benign question environments, 41% of students in our survey perceived themselves to experience moderate to severe stress. Eleven percent of students perceived stress that could adversely affect function. Faculty tended to underestimate the degree of stress students say they experience when questions were used to teach.

Students believed that faculty mostly use questions for evaluation. Faculty stated they mostly use questions to teach. Given the current structure of clinical medical teaching in wards and clinics, it is difficult to separate these two functions of questioning. Furthermore, students’ opinions about whether questions are generally accurate for evaluation differ from their opinions about the accuracy of questions used by faculty to evaluate them as individuals: 86% of students are neutral or agree that questions in general, across the board fairly evaluate knowledge; however, 46% of students felt that their own responses to questions underestimated their knowledge base. This dichotomy may negatively affect the positive learning environment necessary for effective clinical medical education and adds stress to the student faculty interaction [11, 18, 19]. In the high-stakes environment of the medical clerkship, faculty who, with the best of intentions, push with questions to discover the edge of knowledge or to stimulate learning may be pushing some students to greater educational heights but pushing others over the cliff of stress and impairing learning. Faculty may not be able to determine a priori which students are which.

Students (51%) felt much more strongly than faculty (26%) that learning would be enhanced on clinical services if a faculty member’s only role on clinical services was teaching as opposed to evaluation, especially with regard to knowledge base assessment. Overall, 30% of students would prefer no questions or few questions on rounds, perhaps a marker of the potentially hidden degree of stress induced by questioning. This emphasizes the view that asking questions for evaluation negatively impacts learning for some students. Given the pressure that students feel from evaluation, the wide variation in standards both within and across institutions, and the common lack of faculty members’ preparation for their evaluation role [20–22], education leaders should consider whether students have a point about what would best enhance learning on the clinical services. Faculty feedback and development might alleviate some of the learning-impairing stress. However, relieving clinical faculty of the responsibility for summative evaluation of students’ factual knowledge base might reassure students that questions are for learning and therefore enhance learning.

Faculty and students agreed that a malignant question environment has a dramatic negative effect on learning. Forty-six percent of both students and faculty perceived that a malignant question environment at the bedside with a patient present will have the “worst possible effect” on learning. In the past, some faculty or even students have justified a malignant environment on the grounds that aggressive, malignant questioning would incentivize students to learn. Our faculty and students clearly felt that this malignant approach had a detrimental effect on learning and performance.

Many observers have expressed concern about the humiliation caused by “pimping.” In Abbou-Hanna’s study, 39% of students agreed that if students answer a question incorrectly, they feel “humiliated” [2]. In another survey, 74% of students on adult rotations reported experiencing teaching by humiliation [9]. We are troubled by the fact that 40% of those same students considered humiliation “useful for learning” [9]. In one qualitative study, the consensus, as expressed by one student, seemed to be that “You have to kick people’s butts once in a while. Embarrassment is good … because embarrassment kind of motivates people” [11]. In our survey, 13% of students felt that questions used even the hypothetical worst possible learning environment would have a moderately to dramatically *positive* effect on learning. Most students appear to disagree that one must “kick butt” to motivate learning. Certainly, most faculty and students in our survey felt that a malignant question environment had an adverse effect on learning, although a small but meaningful percentage felt it was useful.

A relatively unique finding from our work is that students feel more stress-impaired learning when faculty asked questions with the patient present than when away from the patient in a classroom. Faculty should be cognizant of this additional stress and tailor their approach as appropriate. It may be more difficult to create a “safe” learning environment when the patient is present.

Good reason exists to believe that questions are an effective pedagogical tool. However, questions are not without risk. Some students felt that even benign questions impair their learning and performance. The evaluation role of questions may induce additional stress, and faculty underestimated the degree to which students see questions as evaluation rather than teaching. Performance at the bedside with patients may be especially affected by questions when faculty correct students or students show deficits in knowledge. Most student and faculty respondents see the downside of a malignant question environment. However, a small but meaningful number of students and faculty continue to endorse that strategy.

## Conclusion

For the most part, students and faculty believe questions are useful for learning and evaluation. Attitudes are clearly not uniform. Student and faculty support for or belief in the use of questions is not empiric evidence that questions enhance learning or evaluation in the unique teaching setting of clinical medical education. Some students feel they experience significant stress even in benign environments, and many students certainly feel that questions do not accurately reflect their knowledge or skills, even in a benign question environment. Crucially, faculty often do not recognize the degree of distress that some students feel on clinical rounds, especially in front of patients. Of potential concern, some students and faculty still apparently believe that a malignant question environment enhances learning. Future studies on the actual benefits and risks of using questions to teach and evaluate in the clinical environment should focus on objectively measured learning outcomes and gold standard comparisons for evaluation. Faculty development should be based upon evidence-based techniques and should promote awareness of the differing learning needs of individual students.

## Data Availability

All original questionnaires and online data are available for review.
